# RGD-presenting peptides in amphiphilic and anionic β-sheet hydrogels for improved interactions with cells†

**DOI:** 10.1039/c7ra12503h

**Published:** 2018-03-12

**Authors:** Hodaya Green, Guy Ochbaum, Anna Gitelman-Povimonsky, Ronit Bitton, Hanna Rapaport

**Affiliations:** Avram and Stella Goldstein-Goren Department of Biotechnology Engineering, Ben-Gurion University of the Negev Beer-Sheva 84105 Israel hannarap@bgu.ac.il; Department of Chemical Engineering, Ben-Gurion University of the Negev Beer-Sheva 84105 Israel

## Abstract

The interest in developing functional biomaterials based on designed peptides has been increasing in recent years. The amphiphilic and anionic β-sheet peptide Pro-Asp-(Phe-Asp)_5_-Pro, denoted FD, was previously shown to assemble into a hydrogel that induces adsorption of calcium and phosphate ions and formation of the bone mineral hydroxyapatite. In this study the integrin binding peptide, Arg-Gly-Asp (RGD), was incorporated into the hydrogel to assess its influence on an osteoblast culture. In solutions and in hydrogels FD fibrils dominated the assembly structures for up to 25 mol% FD-RGD incorporation. The cellular density of osteoblasts cultured in hydrogels composed of 25 mol% FD-RGD in FD was higher than that of only FD hydrogel cultures. These results demonstrate that RGD and possibly other cell binding motifs can be combined into amphiphilic and anionic β-sheet hydrogels, using the design principles of FD and FD-RGD systems, to enhance interactions with cells.

## Abstract 2

Schematic presentation of 25% mol FD-RGD in FD anionic β-sheet peptide assemblies (left) that form fibrils (middle). Hydrogels composed of this coassembled peptide system improved cell density compared to FD only hydrogels.

## Graphical abstract

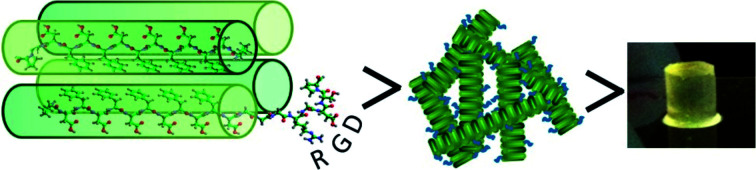

## Introduction

**Fig. 1:**
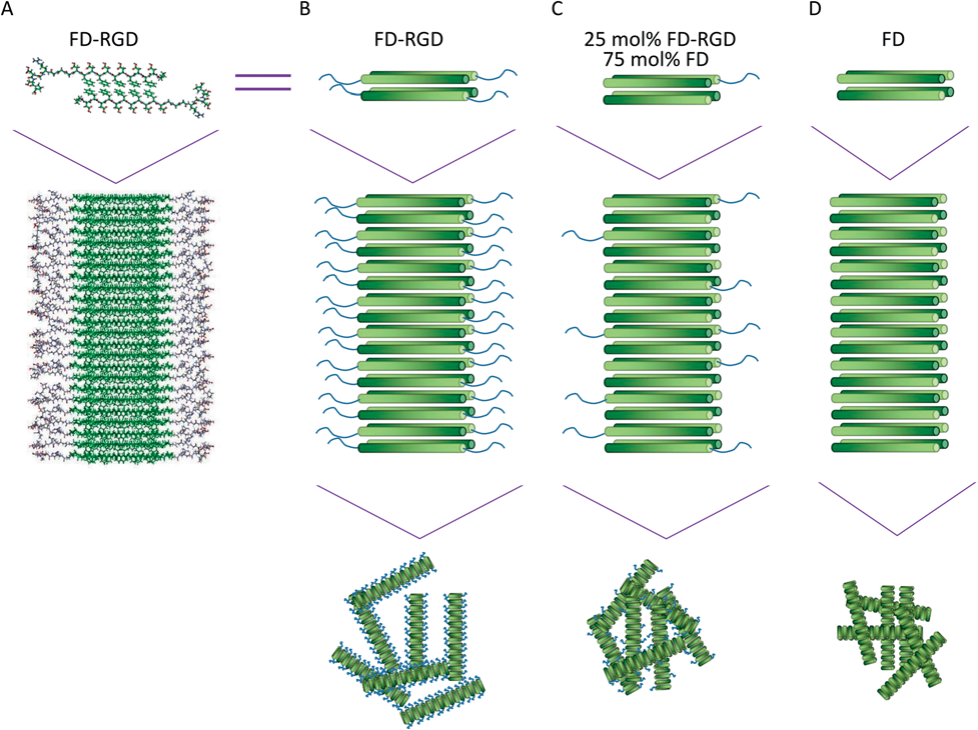
Visualization of FD and FD-RGD assemblies. (A) Molecular models in rod presentation generated in Cerius2 (Accelrys Inc.); two FD-RGD peptides (top) composing the repeat motif of FD-RGD fibril (bottom). Both peptides are drawn with the FD and RGD sections in β-strand and random conformations. The β-strand conformation positions the hydrophobic, Phe side chains from both layers facing each other, while the hydrophilic side chains point to the surrounding aqueous phase. (B–D) The top scheme shows four peptides arranged in a bilayer that constitutes the fibril shown in the bottom scheme. The FD-β-strand section is drawn as a cylinder with a gradient color from the N- to C-termini (dark to bright, respectively) and the RGD-unordered part is depicted as a tail extending from the cylinder C-termini; (B) FD-RGD, (C) 25 mol% FD-RGD and (D) FD.

In recent years there has been an increasing interest in developing functional biomaterials, based on designed β-sheet peptides.^1–7^ In aqueous solutions, amphiphilic peptides with sequences of alternating hydrophobic and hydrophilic amino acids tend to assemble into fibrils composed of β-sheet bilayers. These structures are stabilized by both layers' hydrophilic side chains pointing to the aqueous phase, and hydrophobic side chains zipping the interior of the layers, shielded from the solution (Fig. 1A). Fibrils of β-sheet peptides may stabilize a hydrogel phase under a range of concentrations, pH, ionic strengths or upon other specific intermolecular interactions. β-Sheet hydrogels may provide a synthetic extracellular-like matrix for cell cultures, drug delivery and tissue regeneration applications.^8,9^

The amphiphilic and anionic β-sheet peptide Pro-Asp-(Phe-Asp)_5_-Pro, denoted FD, was shown to form hydrogels that induce calcium-phosphate biomineralization, advantageous to bone tissue regeneration.^10–13^ This peptide hydrogel which is sensitive to pH and calcium concentration^14^ could also be combined with the β-TCP mineral (the β phase of tri-calcium phosphate, Ca_3_(PO_4_)_2_, a medically approved ceramic for treating bone defects) to enhance osteoblastic differentiation and induce faster bone regeneration in rat model.^10^

Scaffolds for tissue regeneration should support cell viability, migration, proliferation and differentiation. One strategy for improving interactions between cells and biomaterials relies on incorporation of peptides with integrin binding sequences that naturally exist in extracellular matrix (ECM) proteins, as fibronectin, laminin and collagen.^15,16^ The peptide Arg-Gly-Asp^17–20^ (denoted RGD) and variations thereof, such as Arg-Gly-Asp-Ser-Pro which is fibronectin's primary binding ligand for the α_5_β_1_ integrin,^21^ were shown to interact with endothelial,^22^ cardiac,^23^ spinal cord,^26^ osteoblastic cells,^24–26^ as well as with other cells types.^27,28^ Such peptides were shown to also improve cell adhesion to polysaccharides as alginate,^29^ and other polymeric matrices such as PHPMA {poly[*N*-(2-hydroxypropyl) methacrylamide]}^25^ and PEG (poly ethylene glycol).^30^

In this study, we were interested in combining the RGD sequence within the peptide matrix to enhance osteoblast interactions with it. The FD-RGD peptide was designed to constitute the FD amino acid sequence, extended with a linker of three Gly residues followed by Arg-Gly-Asp-Ser-Pro. Mixtures of FD and FD-RGD peptides were characterized in solutions by circular dichroism (CD) and in hydrogel phases by cryo-TEM, rheology, SAXS and by evaluating their influence on an osteoblast culture.

## Experimental

### Materials

Pro-Asp-(Phe-Asp)_5_-Pro (denoted FD, *M*_w_ of 1638.67), Pro-Asp-(Phe-Asp)_5_-Gly-Gly-Gly-Arg-Gly-Asp-Ser-Pro (denoted FD-RGD, *M*_w_ of 2225.2) and rhodamine-B N-termini labelled FD were custom synthesized and then purified by high performance liquid chromatography to 95% purity (FD by American Peptide, Sunnyvale, CA, FD-RGD by Genscript, NJ, USA and labelled FD by Caslo, Lyngby, Denmark). Unless otherwise specified, all reagents were purchased from Sigma-Aldrich (Rehovot, Israel) and were of the highest available purity.

### Circular dichroism (CD)

FD and FD-RGD peptide solutions, 0.1 mM, were prepared by dissolving peptide powder in 0.1 mM NaOH (pH = 10) to yield a final pH = 7, followed by sonication for 10 min (Elmasonic S10, Elma, Singen, Germany). Solutions of 25, 40 and 70 mol% FD-RGD were prepared by mixing the 0.1 mM FD and FD-RGD solutions at different volume ratios followed by sonication for a further 20 minutes. The samples were analysed by Circular Dichroism (CD) measurements for mean residue ellipticity, *θ* deg × cm^2^ × dmol^−1^ × number of residues^−1^, in the range of 195–260 nm, at room temperature on a Jasco J-715 spectropolarimeter (Tokyo, Japan), using a 1 mm quartz cuvette. Twenty-four μl of 0.5 M CaCl_2_ were added to 600 μl peptide solution for a final concentration of 20 mM. These samples were sonicated for 10 minutes and CD spectra were analysed again. For mixtures of FD and FD-RGD the average number of residues was used to calculate *θ*.

### FD and FD-RGD mixed peptides hydrogel preparation

Peptide hydrogels were prepared by adding weighed powder of FD or FD and FD-RGD, followed by dissolution in an 80% v/v of the total final solvent (either water, cell culture medium or NaOH 0.1 mM) followed by sonication for 10 min (Elmasonic S10, Elma, Singen, Germany). Next, 20% v/v of CaCl_2_ 0.1 M were added on top of the peptide hydrogel. Samples were then stabilized in a refrigerator overnight prior to characterizations. Hydrogel were prepared at 3.5 to 5% w/v concentrations. These fairly high concentrations generate hydrogels that are stable over a period of at least 3 days and are suitable for cell culture studies.

### Cryogenic transmission electron microscopy (cryo-TEM)

Hydrogels of FD, 25 mol% FD-RGD and FD-RGD, each with overall 5% w/v (corresponding to total molar concentrations of 30, 28 and 22 mM, respectively) were prepared by dissolving in 100 μl NaOH 0.1 mM. Vitrified specimens were prepared on a copper grid coated with a perforated lacy carbon 300 mesh (Ted Pella Inc.). A typically 4 μl from a hydrogel sample was applied to the grid to form a thin film. The samples were immediately plunged into the liquid ethane at its freezing point (−183 °C, Lieca EM GP). The vitrified specimens were transferred into liquid nitrogen for storage. The samples were studied using a FEI Tecnai 12 G2 TEM, at 120 kV with a Gatan cryo-holder maintained at −180 °C, and images were recorded on a slow scan cooled charge-coupled device CCD camera. Images were recorded with the Digital Micrograph software package, at low dose conditions, to minimize electron beam radiation damage.

### Small angle X-ray scattering (SAXS)

5% w/v hydrogels were prepared as described above. Small angle X-ray scattering patterns of peptide solutions were obtained with a SAXSLAB GANESHA 300-XL. CuKα radiation was generated by a Genix 3D Cu-source with an integrated monochromator, 3-pinhole collimation and a two-dimensional Pilatus 300 K detector. The scattering intensity *q* was recorded at intervals of 0.012 < *q* < 0.5 Å^−1^. Measurements were performed under vacuum at the ambient temperature. The scattering curves were corrected for counting time and sample absorption. The solution under study was sealed in thin-walled quartz capillaries about 1.5 mm in diameter and 0.01 mm wall thickness. The scattering spectra of the solvent were subtracted from the corresponding solution data using the Irena package for analysis of small-angle scattering data.^31^ Data analysis was based on fitting the scattering curve to an appropriate model (see ESI†) by software provided by NIST (NIST SANS analysis version 7.0 on IGOR).^32^

### Rheology measurements

600 μl of 5% w/v FD, 10, 15, 25, 40, 70 mol% FD-RGD and FD-RGD hydrogels (corresponding to total molar concentrations of 30, 30, 29, 28, 26 and 22 mM respectively) were prepared as described above (medium as a solvent) and sonicated by probe sonicator (Q125 Sonicator, Qsonica, CT, USA) for 10 s at 20% amplitude. Dynamic frequency sweep rheology measurements were performed on an AR 2000 controlled stress rheometer (TA Instruments, New Castle, DE) operating in cone plate mode with a cone angle of 4° and 2 mm diameter. The elastic moduli of the hydrogels were evaluated by measurement of *G*′ and *G*′′ at a constant stress of 6 Pa as a function of the frequency sweep, *ω* (0.5–100 rad s^−1^). This constant stress value was selected based on strain sweep tests at constant frequency, used to determine the linear viscoelastic region of the hydrogels (see ESI Fig. S1†).

### Hydrogel stability in medium environment

One hundred μl of 5% w/v hydrogels (*n* = 5) of FD, and 10, 15, 25, 40 mol% FD-RGD and FD-RGD hydrogels were prepared as described above with cell growth medium as a solvent (instead of 0.1 mM NaOH). Growth medium contains: DMEM with F-12 (GIBCO, Carlsbad, CA), 10% fetal calf serum (Biological Industries, Beit-Haemek, Israel), 0.3 g L^−1^ gentamicin (GIBCO) and 1% penstrep (Biological Industries). Noteworthy, the medium comes with a concentration of 1 mM calcium ions. Hydrogels were characterized for their overall stability after three days incubation using two parameters: (1) Ca^2+^ concentration remaining within the hydrogel and (2) percentage of peptide released from the hydrogel. The Ca^2+^ concentration in the medium was measured by Atomic Absorption Spectroscopy (Varian, AA240, Palo Alto, CA, USA), and the concentration in the hydrogels was calculated based on the initial Ca^2+^ in these hydrogels that was 20.8 mM. The peptide released from the hydrogels to the serum supplemented medium was measured by absorbance at 258 nm using a microplate-reader (BioTek instruments). The peptide concentration was determined based on a calibration curve of FD peptide prepared in this medium (ESI Fig. S2†).

### Cell culture

For cell culture experiments, h-FOB (human fetal osteoblasts) 1.19 cell line, (ATCC, Manassas, VA) of mesenchymal origin and committed to osteoblast lineage were used. h-FOB 1.19 are immortalized by transfection with temperature sensitive T-antigen mutant (tsA58) of the SV-40 virus undergoing differentiation under restrictive temperature (39.5 °C) when the T antigen is not active, and proliferation at 33.5 °C where the T-antigen is active.^33^ h-FOB were cultured only at 33.5 °C to characterize the effects of FD-RGD peptide during the proliferation stage (avoiding transition to the differentiation stage). Medium was replaced every 3–4 days.

### Cells cultured with 2D peptides hydrogels

One hundred μl of FD and 25 mol% FD-RGD hydrogels were prepared for the cell culture experiment with medium (80% v/v) and supplemented with 0.1 M CaCl_2_ (20% v/v) to reach a final concentration of 20 mM. FD hydrogel was prepared with 3.5% w/v (21 mM) FD, instead of 5% w/v (30 mM) and the 25 mol% FD-RGD was prepared with 21 mM FD and supplemented with 7 mM FD-RGD (3.5% and 1.5% FD and FD-RGD respectively). Prior to cell seeding the hydrogels were placed in 6 well plates, sterilized under UV radiation for 60 min then supplemented with 2 ml of medium and left to stabilize for 60 min at room temperature (the medium was removed after 60 min). Cells were added on two sides of the well (50 000 cells on each side) and covered after 15 minutes with 2 ml of medium. The plates were shaken gently leading to hydrogel disintegration into pieces. One ml of medium was gently replaced after one and three days. After five days, cell viability was measured by live/dead assay applying calcein-AM (green, live) and propidium iodide (red, dead) staining and visualization by fluorescence microscopy at Ex = 490 nm Em = 515 nm and Ex = 535 nm Em = 617 nm respectively. Cells colocalized onto visibly detected hydrogels were measured and represented relative to total live cell area. The stained cultures were observed under a Cool LED pE-2 collimator fitted to an inverted phase-contrast microscope (Eclipse Ti, Nikon) equipped with a digital camera (D5-Qi1Mc, Nikon). For cells and hydrogel quantification, three independent experiments of each condition were analysed. For each sample 4–10 images were taken from different areas in the well and analysed using ImageJ 1.45s software.

### Cell density

An additional 2D hydrogel system that was tested contained 10% (w/w) labelled FD (rhodamine-B, red) peptide. One hundred μM FD (21 mM) and 25 mol% FD-RGD (21 mM FD and 7 mM FD-RGD) labelled hydrogels were prepared with medium (80% v/v) and 0.1 M CaCl_2_ (20% v/v) as described above. The hydrogels were applied on two glass slides (1 × 1 cm^2^; Pgo, Iserlohn, Germany), ∼50 μl each. The hydrogel-glass surfaces were then placed in 24 well plates and sterilized under UV radiation for 60 min. Slides were seeded with a total of 20 000 h-FOB cells and cultured in 1 ml medium for 24 hours. Cells were fixed and stained using Focal Adhesion Staining Kit (FAK100, Millipore) according to the manufacturer's instructions. Vinculin was detected using anti-Vinculin monoclonal antibody and a FITC-conjugated secondary antibody (green) and nuclear counterstaining was done with DAPI (blue). The stained factors were observed under a Cool LED pE-2 collimator fitted to an inverted phase-contrast microscope (Eclipse Ti, Nikon) equipped with a digital camera (D5-Qi1Mc, Nikon). For cells and hydrogel quantification, three independent experiments of each condition were analysed. Following the staining procedure only nuclei could be clearly counted. The area of the gel appearing in red was quantified using ImageJ 1.45s software.

### Cells cultured in 3D hydrogels

FD (100 μl, 21 mM) and 25 mol% FD-RGD (21 mM FD and 7 mM FD-RGD) were prepared with medium (80% v/v) for the 3D cell culture experiment. These were transferred to a 6 well confocal plate, inside the glass area, and then 0.1 M CaCl_2_ (20% v/v) was added. Prior to cell culture the hydrogels were sterilized under UV radiation for 60 min, supplemented with 2 ml of medium and left to stabilize for 60 min at room temperature (the medium was removed after 60 min). h-FOB cells (150 000) were added on top of the hydrogels and cultured for 3 days (1 ml medium replaced after each day). After three days, cells were dyed by live/dead assay, and for the fluorescent image a Spinning-disc confocal microscopy was used {Axiovert-200M microscope (Zeiss, Germany) equipped with Piezo *Z*-Axis head}. Images from each well were taken along 80 μm depth of the hydrogels from the bottom of the well (5–8 points measured for each sample). Live cell volume was calculated by Imaris 8.0.1 program.

## Results

Self-assembly of FD peptides into β-sheet bilayer fibrils can be triggered by various factors including sufficiently high peptide concentration, low pH, positively charged ions and combinations thereof. In order to achieve hydrogels composed of FD that present also the Arg-Gly-Asp-Ser-Pro sequence, a two peptide mixed system was designed. The new peptide FD-RGD was designed (Fig. 1) to facilitate co-assembly with FD into mixed fibrils. In FD-RGD, Pro-Asp-(Phe-Asp)_5_-Gly-Gly-Gly-Arg-Gly-Asp-Ser-Pro, the first twelve residues are identical to those of FD and these are followed by a linker of three Gly amino acids and the fibronectin-derived integrin binding sequence.^34^ In the presumed ideal co-assembly of FD and FD-RGD, with the FD sections of both peptides juxtaposed to satisfy cross-strand H-bonds. As depicted in Fig. 1, the integrin binding motif should protrude out of the fibril to the solution phase.

**Fig. 2:**
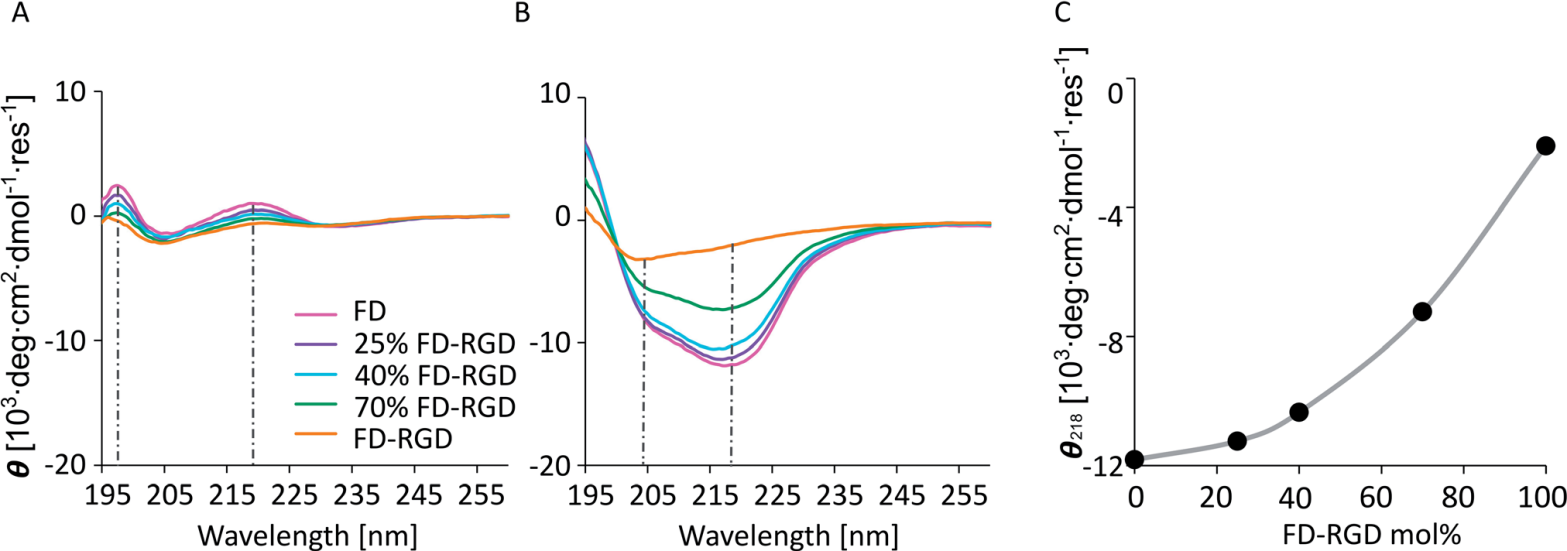
CD spectra, showing mean residue ellipticity, *θ*, *versus* wave length for 0.1 mM peptides solutions with different FD-RGD mol% (A) and with 20 mM Ca^2+^ (B). The dashed grey lines mark the negative peaks at 204 and 218 nm. (C) *θ* at 218 nm (attributed to β-sheet) as function of FD-RGD mol%.

Solutions composed of FD and FD-RGD mixed peptides, at overall concentration of 0.1 mM, pH = 7, characterized by circular dichroism (CD, Fig. 2) showed similar absorption patterns for all the tested samples, FD, 25, 40, 70 mol% FD-RGD and FD-RGD, with two positive peaks at 198 and 220 nm and a shallow negative peak at 204 nm (Fig. 2A). Such positive CD peaks, associated with stacking interactions between Phe side chains, were previously detected also by us for the peptide Phe-Glu-Phe at low peptide concentrations.^35–37^ At higher peptide concentrations the CD spectra of this tripeptide transformed into the commonly detected spectra of β-sheet structures. Hence, CD spectra of FD and FD-RGD and their mixtures, exhibit similar conformations in solution, that enable coexisting Phe–Phe interactions along with generally unordered conformation, attributed to the negative absorption peak at 204 nm, that is expected at low concentration regions of such negatively charged peptides.

On addition of calcium chloride solution all spectra switched to showing two negative absorption peaks with minima at 218 and 204 nm, associated with β-sheet and the unordered conformations, respectively (Fig. 2B). The calcium is responsible for inducing the formation of β-sheet structures, by counterbalancing the anionic charge on the peptide and enabling their assembly into fibrils. In order to assess the effect of FD-RGD content on the tendency of the mixed peptide system to form β-sheet structures, we plotted the CD absorption minima at 218 nm as function of FD-RGD mol% fraction (Fig. 2C). This plot reveals that in mixtures of the two peptides with low FD-RGD content (up to 40 mol%) the β-sheet structure induced by FD prevails. Since the trend of 218 absorption is non-linear with the change in concentration it can be deduced that FD-RGD incorporates into FD fibrils while adopting the β-sheet conformation (as represented schematically in Fig. 1C). At higher FD-RGD content similarly to pure FD-RGD there is lower tendency for β-sheet formation (note deeper absorption of the unordered conformation at 204 nm, compared to that at 218 nm). Therefore, the schematic structure of the FD-RGD fibril, depicted in Fig. 1D, is less favourable than that of FD.

**Fig. 3:**
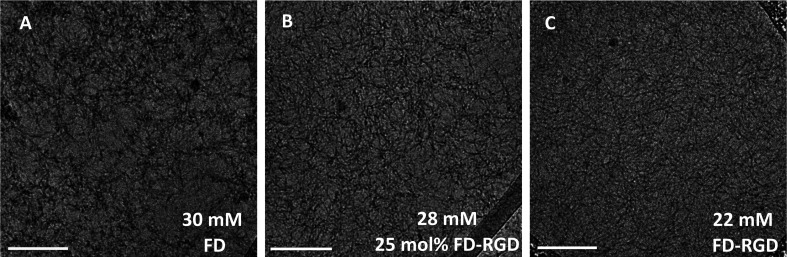
cryo-TEM images of 5% w/v hydrogels of FD, 25 mol% FD-RGD and FD-RGD (equivalent to 30, 28 and 22 mM, respectively). Scale bar = 200 nm. Fibrils average thickness based on image analysis (see ESI† S3) are ∼8 nm for FD and 25 mol% FD-RGD and ∼6 nm for FD-RGD. ESI S4† shows TEM images of 0.1 mM (0.02% w/v) solutions, as measured by CD above, demonstrating lower extent of fibrils that do not generate hydrogels.

**Fig. 4:**
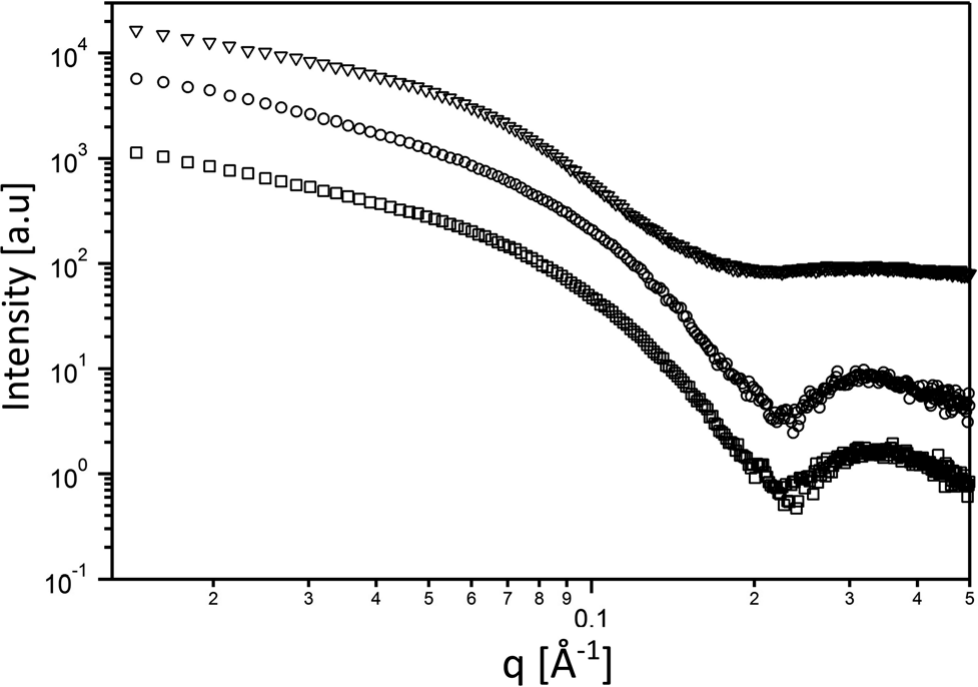
Small angle scattering curves of the 5% w/v hydrogels. FD (○) 25 mol% FD-RGD (□), and FD-RGD (Δ).

Next, we aimed at elucidating the effect of FD-RGD content on the mechanical properties of hydrogels prepared with FD and FD-RGD mixtures. At overall weight concentration of 5% w/v both peptides and their mixtures, when supplemented with 20 mM calcium chloride form self-supporting hydrogels. Here we note that based on previous research^12,14^ hydrogel formation is also possible in a range of concentrations, >1% w/v, without calcium yet it has been shown that higher peptide concentrations and added calcium enhance the hydrogels stability. cryo-TEM images (Fig. 3) of samples of these FD, 25 mol% FD-RGD and FD-RGD hydrogels showed in general fibril networks (the hydrogels form immediately). The images revealed that FD appears with denser fibril loci compared to the 25 mol% FD-RGD mixture whereas pure FD-RGD shows lower density and dispersed thin fibrils (see ESI Fig. S3†). These results imply that FD fibrils may coalesce into thicker fibril aggregates and this tendency is hindered by the incorporation of FD-RGD possibly due to steric interference of RGD tails protruding out of the fibrils (as shown in Fig. 1C). Small angle X-ray scattering (SAXS) spectra (Fig. 4) were also applied on these hydrogels. The FD spectrum exhibits a peak at *q*_0_ ∼ 0.33 Å^−1^ suggesting structures with a characteristic length of ∼18 Å (calculated by 2π/*q*_0_) in accordance with the fibril bilayer thickness (demonstrated in Fig. 1). The scattering curve of 25 mol% FD-RGD hydrogel shows a slightly weaker peak at *q*_0_ ∼ 0.33 Å^−1^, indicating that FD-RGD had little effect on the extent of fibril formation in accordance with the CD measurements at low FD-RGD content. In the scattering curve of the FD-RGD hydrogel, this peak is hardly visible indicating that a much lower concentration of fibril structures is formed by this peptide, again in accordance with CD measurements.

The spectrum of FD hydrogel was also fitted to a core–shell cylinder form factor (see ESI Fig. S5†) similar to that applied in previously reported modelling of amphiphilic lipopeptide assemblies.^38^ It should be noted that in order to fit the scattering of the FD-RGD to the model a subtraction of the background was conducted. As expected all three curves could be fitted to the same model yielding a core radius of 17 Å ± 2, and a shell thickness 18 ± 2 Å.

**Fig. 5:**
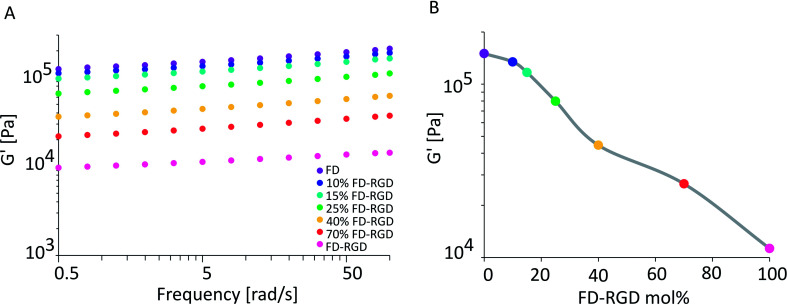
(A) Elastic modulus *G*′ obtained by rheology measurements, of 5% w/v hydrogels with different FD-RGD mol%, dissolved in cell culture medium and supplemented with 20 mM Ca^2+^ ions. (B) *G*′ at frequency 5 [rad s^−1^] of the hydrogels as function of FD-RGD mol% (grey line for guiding the eye).

Rheology measurements were applied to 5% w/v hydrogels composed of FD, 10, 15, 25, 40, 70 mol% FD-RGD and FD-RGD, prepared with cell culture medium (see Experimental) to be relevant for cell culture studies described hereafter. All mixtures formed self-supporting hydrogels that in sweep mode constant stress mode, exhibited storage moduli, *G*′ (Fig. 5), higher than the loss moduli *G*′′ (data not shown) over the shear rates 0.5–100 rad s^−1^. Interestingly, *G*′ as a function of FD-RGD percentage shows apparently two zones. At high FD and low FD.

RGD (up to 25 mol%) content there is a weak decrease in *G*′ as function of FD-RGD, implying dominance of the stronger FD hydrogel mesh. Beyond 40 mol% FD-RGD, there is a linear decline in the hydrogel strength as function of increased FD-RGD content.

The same type of hydrogels prepared with cell culture media (as above) were assessed for their dissolution into medium. Hydrogels were prepared within syringes, covered with medium four times their volume and after 72 hours of incubation peptide and calcium concentrations were measured in the medium phase. Following the 72 hour incubation time in the syringe both FD and FD-RGD hydrogels maintained their apparent macroscopic structure and peptide concentrations released from the hydrogels into the medium were very low, less than 3% (Table 1). There were minor differences in calcium concentrations found in the different hydrogels post incubation. In the FD hydrogel calcium concentrations within the hydrogel were found to be slightly higher than the initial concentration (20.8 mM, used for preparing all hydrogels), as a result of absorption from the medium. Noteworthy, Ca^2+^ concentration used for preparing hydrogels relative to total peptide concentration (ranging in molar concentrations between 30 for FD, to 22 mM for FD-RGD) does not provide full charge neutralization of the anionic Asp residues; full neutralization of FD would require a molar ratio of 2 between peptide anionic residues to Ca^2+^. Under the conditions of the experiment here, the mole ratio between anionic groups in hydrogels relative to calcium ions was found to be 8.1–7.6 (Table 1), indicating equilibration of the hydrogel with partial neutralization of the anionic residues by the calcium ions, in accordance with our previously reported FD–calcium equilibrium studies in Tris buffer saline.^10^

**Table 1:** Dissolution assay of FD and FD-RGD mixed peptide hydrogels. Both FD and FD-RGD were prepared as 5% w/v stock solutions from which mixtures were prepared. Peptide and calcium concentrations were measured post 72 hours of incubation (*n* = 5, see ESI Table 1)

Hydrogel type mol%	Mol% peptide in hydrogels	Mol% Ca^2+^ in hydrogels	Anionic residues in hydrogel/Ca^2+^ mol ratio
FD	97	105^a^	8.1
10% FD-RGD	97	103	8.3
15% FD-RGD	97	101	8.4
25% FD-RGD	97	100	7.9
40% FD-RGD	99	97	7.8
FD-RGD	99	85	7.6

a The concentration after incubation is higher than the initial since there is adsorption of Ca^2+^ ions from the medium.

**Fig. 6:**
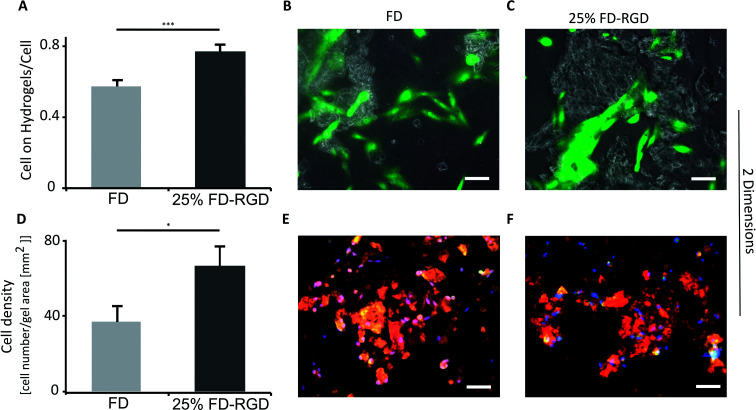
Live h-FOB cells area (calcein-AM assay) cultured on 2D peptide hydrogels after 5 days. (A) Ratio between areas of live cells on FD or 25 mol% FD-RGD hydrogel to total live cells area, ***p < 0.001, *n* = 3. Images of cells stained live (green) cultured on (B) FD hydrogel and on (C) 25 mol% FD-RGD. (D) Number of cells stained by DAPI (blue, as part of Focal Adhesion Staining Kit, see Experimental) relative to hydrogel area of FD and 25 mol% FD-RGD peptide hydrogels labelled with fluorescent rhodamine-B (red), after 24 h culture, *p < 0.05, *n* = 3. Yellow which indicates merge between red (hydrogel) and green (staining vinculin by the kit) was not used for analysis due to low contrast between the different shades of the red hydrogels and the yellow colour. (E) Images of cells over FD and (F) over 25 mol% FD-RGD. Scale bar = 100 μm, two-tail student test, SEM in errors bars. Noteworthy, there was no significant difference in overall cell viability between FD and 25 mol% FD-RGD (see ESI S6†).

**Fig. 7:**
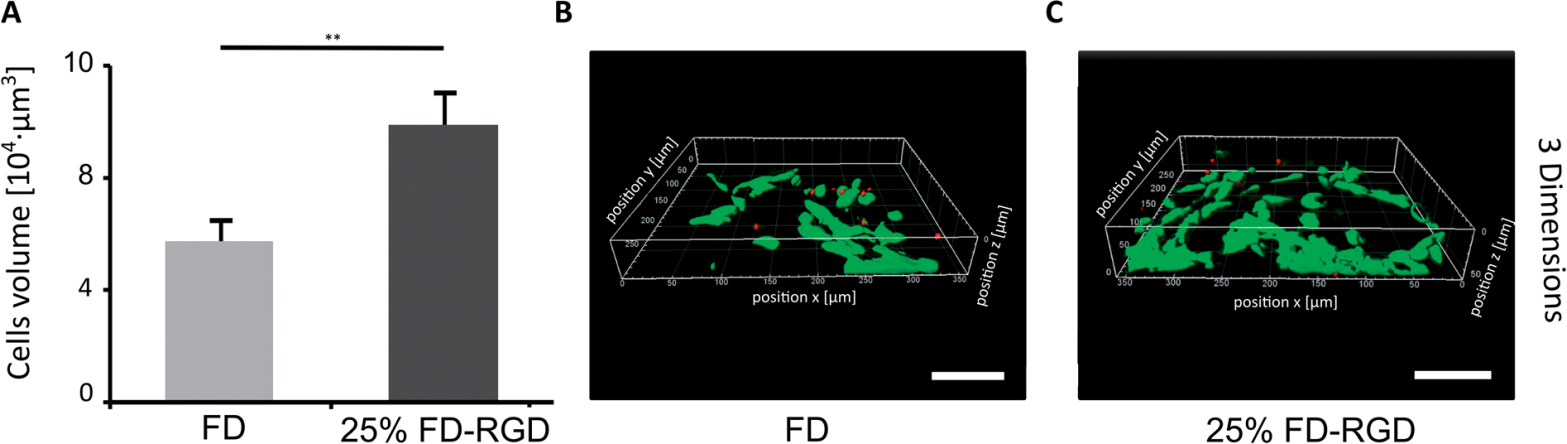
h-FOB cells cultured with 3D peptide hydrogels, stained with live/dead assay after 3 days in 3D system. (A) Live cell volume calculated based on confocal images. (B) Image of cell culture with FD or (C) 25 mol% FD-RGD. Scale bar = 100 μm, two-tail student test. Error bars represent SEM, **p < 0.01 (*n* = 3).

Osteoblasts viability was evaluated in 2D and 3D cell culture systems (Fig. 6 and 7) in two types of hydrogels having similar *G*′ value so to minimize influence of rheological properties on cell behaviour. The hydrogels selected for these assays were composed of 3.5% w/v FD, equivalent to 21 mM, and of 5% w/v FD and FD-RGD mixed hydrogel prepared with 21 mM FD and 7 mM FD-RGD, denoted 25 mol% FD-RGD. Noteworthy, the lower peptide concentration used for preparing the FD hydrogel was necessary for obtaining clear hydrogels amenable to light microscopy assays applied hereafter. A 2D growth system was obtained by placing 100 μl hydrogel in a six well plate that was covered with 2 ml cell culture medium. In each well 100 000 h-FOB cells were seeded and cultured for 5 days. Following this period of time the hydrogels underwent fragmentation and by light microscopy pieces of the hydrogel could be detected along with the cells. Live/dead assay applied to these cultures with calcein-AM (staining green live cells) and propidium iodide (staining red dead cells), showed that on the 25 mol% FD-RGD hydrogel 77 ± 18% live cells were found whereas on FD significantly fewer live cells, 57 ± 18%, were observed (Fig. 6A). In an additional 2D culture system, the hydrogels were stained with FD-rhodamine-B labelled peptide (see Experimental) so to enable better visualization of the hydrogel pieces. Over cover glasses, 50 μl of hydrogels were spread, then seeded with 20 000 h-FOB cells and cultured for 24 h. The whole system was then subjected to staining with DAPI (staining nuclei in blue) and the number of nuclei appearing over the red stained hydrogel was counted. Fig. 6D shows a higher number, 61.3 ± 9.2 cells per mm^2^ hydrogel area, co-localized with the 25 mol% FD-RGD hydrogels compared to 34.4 ± 6.5 cells per mm^2^ hydrogel area of FD (Fig. 6D–F).

A 3D cell culture system (Fig. 7) was prepared with 100 μl hydrogels placed in confocal wells (see Experimental). h-FOB cells (150 000) were seeded on hydrogels and cultured for three days. Then live/dead assay was applied and images of cells were acquired by inverted confocal microscope, which allowed imaging at 80 μm distance from the bottom of the hydrogel (hydrogel total depth was ∼1000 μm). The volume of live cells sampled within this depth (Fig. 7A) was found to be almost two-fold higher in the 25 mol% FD-RGD system (99 000 ± 6000 μm^3^, Fig. 7C) than the volume of live cells in FD hydrogel (57 000 ± 7000 μm^3^, Fig. 7B).

## Discussion

FD peptide which was previously shown to form hydrogels that support osteoblast growth and induce bone regeneration was combined in this study with a newly designed peptide FD-RGD, to generate hydrogels presenting the Arg-Gly-Asp-Ser-Pro motif that facilitates cell adhesion through binding with integrin receptors. FD organizes into a β-sheet secondary structure that forms fibrils in solution, which at sufficiently high concentrations stabilizes in a hydrogel phase. FD-RGD was designed based on the assumption that it would intercalate into FD fibrils while leaving the Arg-Gly-Asp-Ser-Pro residues protruding out of the fibril structure to enable interactions with the cell membrane receptors.

FD and FD-RGD mixtures studied in solution by CD, showed similar absorption spectra corresponding to random peptide conformation coexisting with Phe–Phe stacking interactions. Since both pure FD and FD-RGD showed similar CD patterns it cannot be deduced whether the peptides segregate or form a well-mixed system. Yet, following the addition of calcium ions the CD spectra revealed differences between FD and FD-RGD with FD giving a strong β-sheet structure signal, that became weaker with the addition of FD-RGD to the mixture. The β-sheet absorption formed by the two peptides indicated high β-sheet content similar to that of pure FD, for up to 25 mol% FD-RGD followed by a linear decrease with higher FD-RGD content. The non-linear dependence on FD-RGD content at its low concentrations, may point to the dominance of the FD β-sheet assembly and the intercalation of FD-RGD in between FD fibrils, as demonstrated in Fig. 1. At higher FD-RGD with linear decrease in β sheet formation as function of this peptide content, it is possible that the two peptides segregate to two different fibril types. cryo-TEM images samples of FD hydrogel showed regions with condensed fibrils meshes and FD-RGD appeared composed of finely dispersed thin fibrils. Hydrogel fibrils of 25 mol% FD-RGD appeared more similar those of FD in accordance with the dominance of FD assemblies detected in CD measurements up to 25 mol% FD-RGD.

Rheology measurements of FD and FD-RGD mixtures indicated that FD forms stiffer hydrogels than FD-RGD. This result implies that FD-RGD on its own has lower tendency to form fibrils and possibly these fibrils have lower tendency to form inter-fibril junction points believed to contribute to the hydrogel stiffness. These differences between FD and FD-RGD fibrils may stem from differences in fibril termini rigidity. FD fibril termini are composed of rigid Pro residues held by neighbouring residues in β-sheet conformation (Fig. 1C) whereas in FD-RGD the termini are not expected to be in β-sheet conformation since the alternating hydrophobic-hydrophilic motif is not maintained along the tail of Gly-Gly-Gly-Arg-Gly-Asp-Pro. As a consequence, these fibrils show no preference for fibril–fibril interactions resulting in a fairly weak hydrogel structure. Indeed, TEM images demonstrated bundles of interacting fibrils for FD and dispersed non interacting fibrils to FD-RGD. Interestingly, at low FD-RGD content, up to 25 mol%, rheology measurements showed a minor (non-linear) decrease in *G*′ pointing to dominance of FD fibril structures in the mixed fibrils as demonstrated in Fig. 1B.

FD and FD-RGD hydrogels in general reached equilibrium similar to that we previously reported for FD^10^ with calcium concentrations ∼20 times higher in hydrogels relative to the surrounding, which in cell culture medium is ∼1 mM. Mixed FD and 40 mol% FD-RGD showed dissolution behaviour similar to that of FD in agreement with the envisioned similarities in the packing models of FD and FD-RGD, where both are held by cross-strand interactions between the FD section repeats.

For cell binding studies, 25 mol% FD-RGD mixed hydrogels were chosen so to exhibit the dominance of FD fibrils intercalated with FD-RGD peptides. In such a composition of mixed hydrogels the influence of Arg-Gly-Asp-Ser-Pro motif on osteoblast adhesion was expressed in 35% more cell area and nearly double the density of cells over the 25 mol% FD-RGD compared to FD. Almost double the cell volume was also found in 3D cultures of FD-RGD hydrogels compared to FD peptide hydrogels. These results demonstrate that cell interactions with FD hydrogels can be further improved with the incorporation of peptides constructed as FD-RGD in which an FD motif is extended by a cell binding motif. This study may assist in improving the design of complex multifunctional peptide assemblies for interactions with different cell types.

## Conclusions

The hydrogels combined of both FD-RGD and FD potentially present an improved bone regeneration matrix. The evidence that RGD is active in FD dominated mixtures supports the applicability of this dual peptide system design for additional cell binding sequences.

## Conflicts of interest

There are no conflicts to declare.

## Supplementary Material

RA-008-C7RA12503H-s001
