# Supplementary material for: RGD-presenting peptides in amphiphilic and anionic β-sheet hydrogels for improved interactions with cells
Source: RSC Adv. 2018 Mar 12;8(18):10072–80. doi: 10.1039/c7ra12503h (PMC9078708; doi:10.1039/c7ra12503h)
Supplement: RA-008-C7RA12503H-s001 [file RA-008-C7RA12503H-s001.pdf]

Electronic Supplementary Material (ESI) for RSC Advances.
This journal is © The Royal Society of Chemistry 2018

Supplementary Information

RGD-presenting peptides in amphiphilic and anionic β-sheet hydrogels for improved interactions with cells

Hodaya Green, Guy Ochbaum, Anna Gitelman Povimonsky, Ronit Bitton, Hanna Rapaport

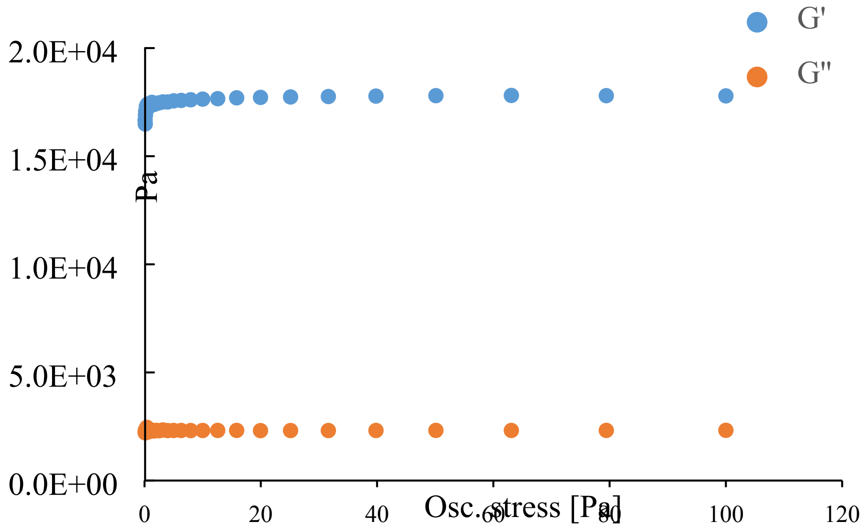

Figure S1: Strain sweep test at constant frequency used to determine the linear viscoelastic region of FD-RGD hydrogel.

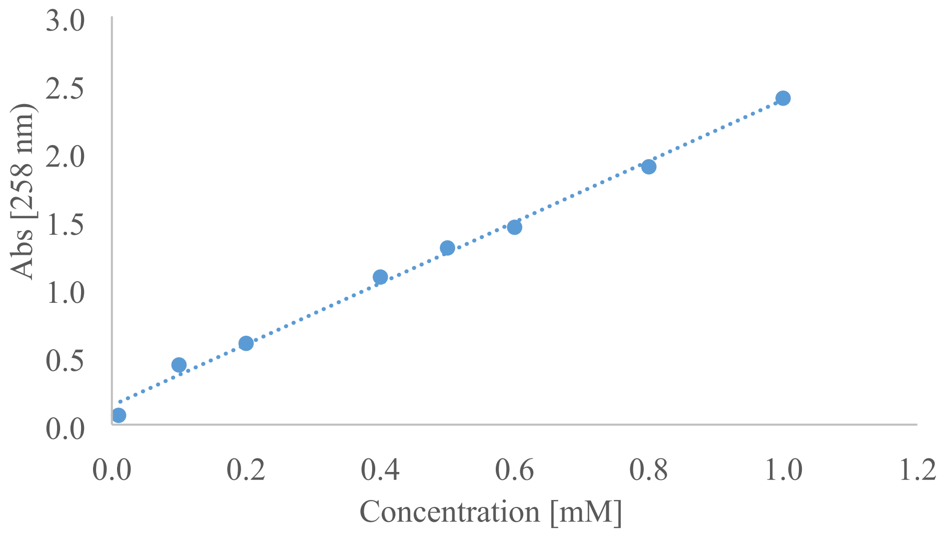

Figure S2: calibration curve of FD peptide prepared in this medium.

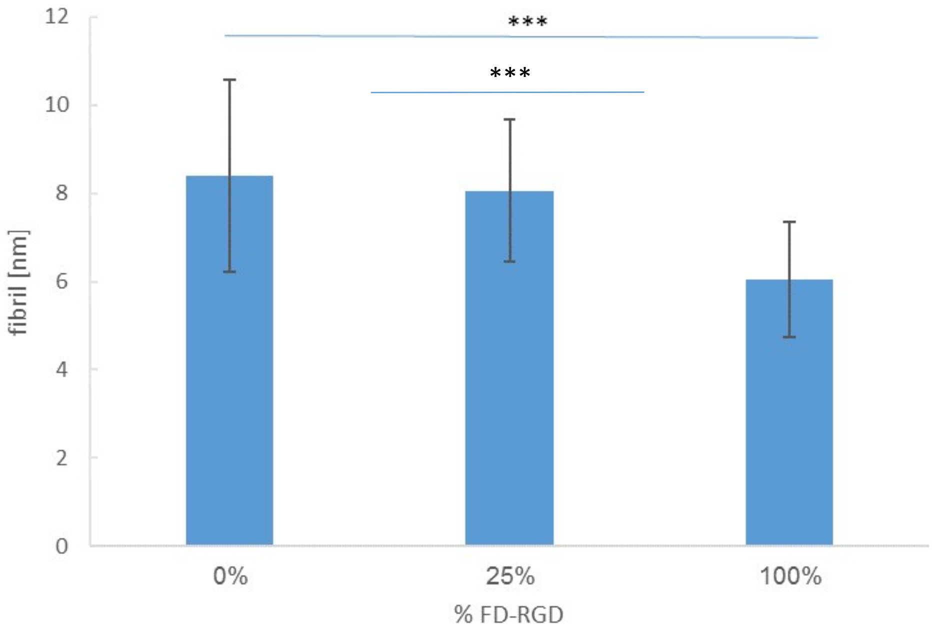

Figure S3: Mean fibrils thickness in different hydrogels (based on Figure 3 cryo-TEM images). Fibrils thickness was calculated using the ImageJ software for 3 TEM images and 20 fibrils in each image. Statistical analysis by T-test *** $p < 0.001$, error bar- standard deviation**.**

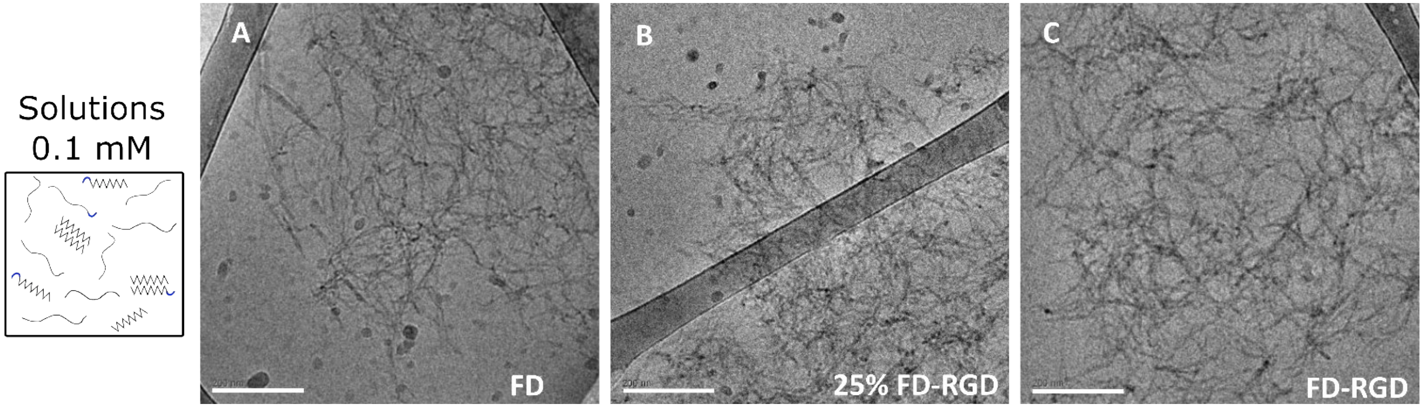

Figure S4: TEM images of 0.1 mM (0.02 % w/v) solutions demonstrating lower extent of fibrils that do not generate hydrogels.

Modeling of small-angle scattering patterns

Core Shell Cylinder

FD can be described by a form factor for a core-shell cylinder given by:

$$p(q) = \frac{scale}{V_{shell}} \int_{0}^{\pi/2} f^2(q,\alpha)\sin\alpha d\alpha \qquad (e.q\ 1)$$

$$f(q,\alpha) = 2(\rho_{core} - \rho_{shell})V_{core}J_0(qH\cos\alpha)\frac{J_1(qr\sin\alpha)}{qr\sin\alpha} +$$

$$2(\rho_{shell} - \rho_{solvent})V_{shell}J_0[(qH+t)\cos\alpha]\frac{J_1(q(r+r)\sin\alpha)}{q(r+t)\sin\alpha}$$

Where r is the radius of the core of the cylinder, and $J_1(x)$ is the first order bessel function. Alpha is defined as the angle between the cylinder axis and the scattering vector, q.

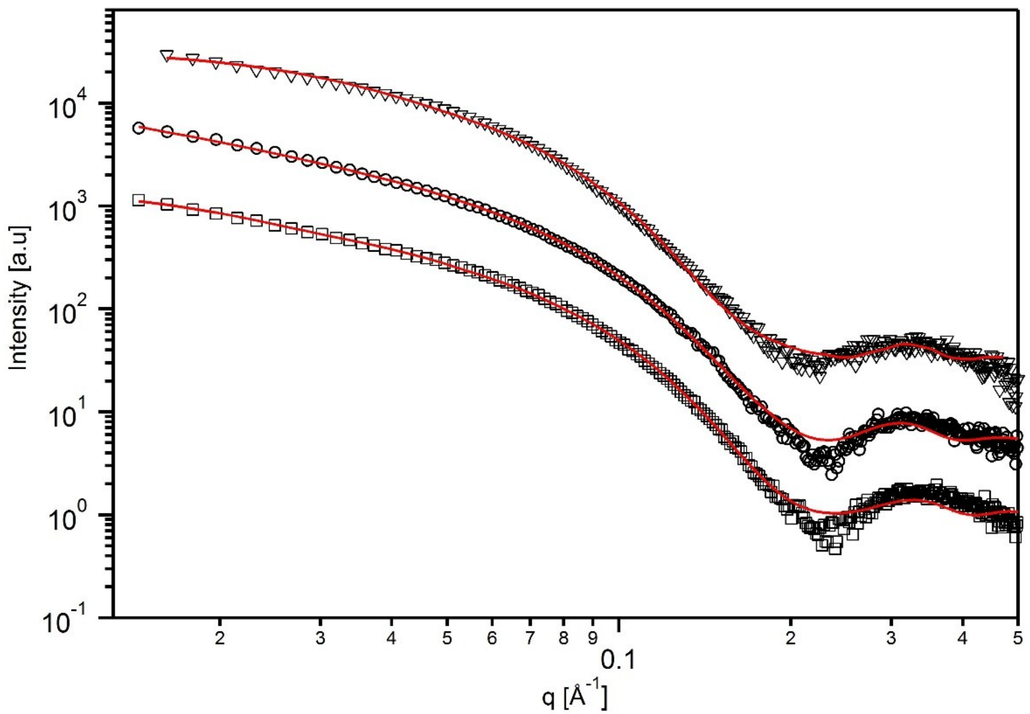

Figure S5. Small angle scattering curves of the 5% w/v hydrogels. FD (○) 25 mol% FD-RGD (□), and FD-RGD (∆). The solid black lines represent fits to the core-shell cylinder model. The best fit parameters were: core radius 17 Å, and shell thickness 18 Å. The solid red lines represent fits to the model described by eq.1 .The curves are shifted to facilitate better visualization.

Table S1 Dissolution assay of FD and FD-RGD mixed peptide hydrogels. Both FD and FD-RGD were prepared as 5% w/v stock solutions from which mixtures were prepared. Peptide and calcium concentrations were measured post 72 hours of incubation (n =5)

Theoretical composition of mixed hydrogels					Measured concentrations in prepared hydrogels					
weight % FD-RGD	weight % FD	mM FD-RGD	mM FD	Total initial peptides conc. mM	Measured peptides conc. before incubation mM	Measured peptides conc. after incubation mM	% peptides in hydrogel after incubation	Measured Ca^{+2} conc. after incubation mM	% Ca^{+2} after incubation	anionic groups in hydrogel/Ca^{+2} after incubation
	5		30.5	30.5	30.5	29.5	96.7	21.8	105	8.1
0.5	4.5	2.2	27.5	29.7	30.7	29.9	97.4	21.5	103	8.3
1	4	4.5	24.4	28.9	30.4	29.5	97.0	21	101	8.4
1.5	3.5	6.7	21.4	28.1	28.3	27.5	97.2	20.8	100	7.9
2.5	2.5	11.2	15.3	26.5	26.5	26.2	98.9	20.2	97	7.8
5	0	22.5	0	22.5	22.5	22.2	98.7	17.6	85	7.6

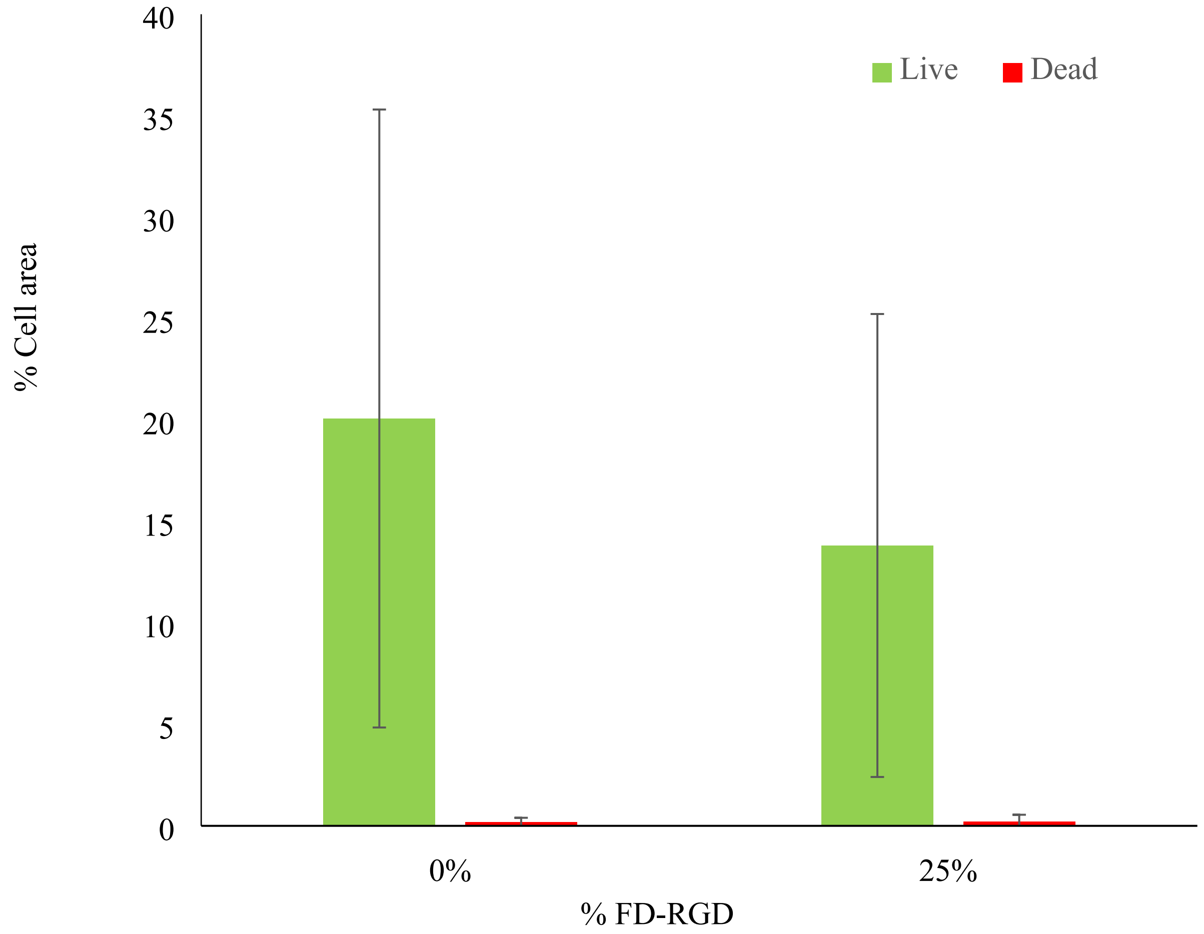

Figure S6. h-Fob cell line viability in FD hydrogel and 25 mol% FD-RGD hydrogel. Green bars- average of live cells in image and red bars- average of dead cells area in image. , there was no significant difference in overall cell viability between FD and 25 mol% FD-RGD.
